# α-Glucosidase inhibitors from the bark of *Mangifera mekongensis*

**DOI:** 10.1186/s13065-016-0193-9

**Published:** 2016-07-21

**Authors:** Hai Xuan Nguyen, Tri Cong Le, Truong Nhat Van Do, Tho Huu Le, Nhan Trung Nguyen, Mai Thanh Thi Nguyen

**Affiliations:** Faculty of Chemistry, University of Science, Vietnam National University Hochiminh City, 227 Nguyen Van Cu, District 5, Hochiminh City, Vietnam; Cancer Research Laboratory, Vietnam National University Hochiminh City, 227 Nguyen Van Cu, District 5, Hochiminh City, Vietnam

**Keywords:** *Mangifera mekongensis*, Anacardiaceae, α-Glucosidase inhibition, Sterols

## Abstract

**Background:**

*Mangifera mekongensis* (Anacardiaceae) is cultivated for its edible fruit and has been used in traditional Vietnamese medicine for its anti-aging properties and for treating diabetes, vermifuge, and dysentery. As part of a search for biologically active compounds with reduction of the rate of glucose absorption, a screening has been initiated to evaluate natural product extracts for the inhibition of enzyme α-glucosidase. A *n*-hexane extract of the bark of *M. mekongensis* showed strong α-glucosidase inhibitory activity with IC_50_ value of 1.71 µg/mL. Thus, the constituents of this plant were examined.

**Results:**

Two new steroids named mekongsterol A (**1**) and mekongsterol B (**2**), were isolated from the *n*-hexane extract of the bark of *M. mekongensis* (Anacardiaceae), together with seven known compounds (**3**–**9**). Their chemical structures were elucidated on the basis of spectroscopic data. All compounds possessed significant α-glucosidase inhibitory activity in a concentration-dependent manner, except for **3** and **4**. Compounds **1**, **2**, **5**–**9** showed more potent inhibitory activity, with IC_50_ values ranging from 1.2 to 112.0 µM, than that of a positive control acarbose (IC_50_, 214.5 µM).

**Conclusions:**

These results suggested that the traditional use of the bark of *M. mekongensis* for the treatment of diabetes diseases in Vietnam may be attributable to the α-glucosidase inhibitory activity of its steroid and cycloartane constituents.

**Electronic supplementary material:**

The online version of this article (doi:10.1186/s13065-016-0193-9) contains supplementary material, which is available to authorized users.

## Background

**Fig. 1:**
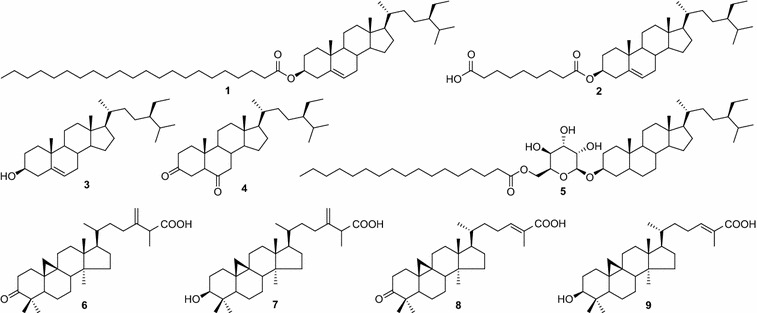
Structures of the isolated compounds from the bark of *M. mekongensis*

*Mangifera mekongensis* (Anacardiaceae), commonly known as mango, is widely distributed in tropical and subtropical regions of Asia. In Vietnam, *M. mekongensis* is called as “Xoai Thanh Ca”, and this plant is cultivated for its edible fruit and has been used in traditional Vietnamese medicine for treating anti-aging, diabetes, vermifuge, dysentery [1, 2]. A research for biologically active compounds with reduction of the rate of glucose absorption, a screening has been initiated to evaluate natural product extracts for the inhibition of enzyme α-glucosidase. It is effective in controlling postprandial hyperglycaemia and prevents complications associated with type-II diabetes, such as microvascular (i.e., retinal, renal, and possibly neuropathic), macrovascular (i.e., coronary and peripheral vascular), and neuropathic (i.e., autonomic and peripheral) complications [3, 4]. Previously, we reported that the methanolic extracts of *Embelia ribes*, *Oroxylum indicum*, and *Artocarpus altilis* exhibited significant inhibitory activity on α-glucosidase [5–8]. In a part of our continued research on the screening of medicinal plants of different origins, we also found that the *n*-hexane extract of the bark of *M. mekongensis* showed strong α-glucosidase inhibitory activity with IC_50_ value of 1.71 µg/mL. Thus, we carried out the bioactivity-guided fractionation of *n*-hexane extract of this plant and isolated two new steroids, mekongsterols A (**1**) and B (**2**), together with seven known compounds (**3**–**9**) (Fig. 1). In this paper, we describe the isolation and structural elucidation of these compounds by spectroscopic methods as well as their α-glucosidase inhibitory activity.

## Result and discussion

### Chemistry

The dried powdered bark of *M. mekongensis* was extracted with *n*-hexane in Soxhlet extractor to yield *n*-hexane fraction. Further separation and purification of this fraction led to the isolation of two new steroids, mekongsterols A (**1**) and B (**2**), together with seven known compounds (**3**–**9**). The known compounds were identified by the analysis of their spectroscopy data and comparing with the literature data to be as β-sitosterol (**3**) [9], stigmastane-3,6-dione (**4**) [10], β-sitosteryl-3-*O*-β-D-glucopyranosyl-6′-*O*-palmitate (**5**) [11], mangiferonic acid (**6**) [12], mangiferolic acid (**7**) [12], ambonic acid (**8**) [13], and ambolic acid (**9**) [12] (Fig. 1).

**Fig. 2:**
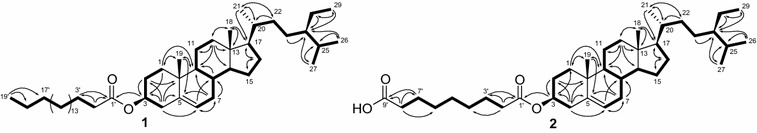
Connectivity (*bold lines*) deduced by the ^1^H-^1^H Correlation Spectroscopy (COSY) spectrum and significant HMBC correlations (*arrows*) observed for **1** and **2**

**Fig. 3:**
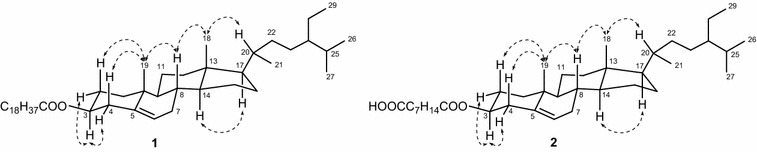
Key NOESY correlations observed for compounds **1** and **2**

Mekongsterol A (**1**) was obtained as a white crystal and showed the quasimolecular ion at *m*/*z* 733.6223 [M + K]^+^, corresponding to the molecular formula C_48_H_86_O_2_K in HR-ESI–MS. The IR spectrum of **1** showed absorption of ester carbonyl (1720 cm^−1^), double bond (1610 cm^−1^), and methyl, methylene, and methine (2950 and 2870 cm^−1^) groups. The ^1^H NMR spectrum of **1** (Table 1) displayed signals due to two methyl singlets (*δ*_H_ 0.68, 1.02, each s), three methyl doublets (*δ*_H_ 0.81, d, *J* = 6.8 Hz; *δ*_H_ 0.84, d, *J* = 6.8 Hz; *δ*_H_ 0.92, d, *J* = 6.5 Hz), a methyl triplet (*δ*_H_ 0.82, t, *J* = 7.5 Hz), an oxymethine (*δ*_H_ 4.62, m), and trisubstituted olefinic bond (*δ*_H_ 5.38, d, *J* = 4.4 Hz), together with many aliphatic methylene and aliphatic methine groups (*δ*_H_ 0.95–2.30). The ^13^C NMR (Table 1) and DEPT spectra of **1** exhibited signals for six methyls (*δ*_C_ 12.0, 12.1, 18.9, 19.2, 19.5, 19.9), an oxymethine (*δ*_C_ 73.8), and two olefinic carbons (*δ*_C_ 122.7 and 139.9). These data closely resembled those of β-sitosterol (**3**) [9], a common steroid found in plants, but they were characterized by the presence of additional signals due to a saturated fatty ester chain having 19C, which showed ester carbonyl (*δ*_C_ 173.5), many methylenes (*δ*_H_ 1.20–2.27; *δ*_C_ 22.8–34.9), and one methyl triplet (*δ*_H_ 0.88, t, *J* = 6.9 Hz). The location of saturated fatty ester chain was determined to be at C-3 on the basis of the low-field shift of H-3 (*δ*_H_ 4.62) compared to that of **3** (*δ*_H_ 3.51), which was confirmed by the HMBC correlation from H-3 to C-1′ (Fig. 2). The orientation of saturated fatty ester group at C-3 was determined β-equatorial from the NOESY correlations H-3/H-2α and H-3/H-4α, and large *J* value (7.7 Hz) between H-3 and H-4β (Fig. 3). The relative stereochemistry of **1** was assigned on the basis of NOESY correlations and coupling constant data. The NOESY correlations H-3/H-4α, H-3/H-2α, H-14/H-17, H-2β/H_3_-19, H-4β/H-19, H-19/H-8, H-8/H_3_-18, and H_3_-18/H-20, together with the large coupling constant (*J* = 11.9) between H-8 and H-14 suggested that rings C and D to be *trans*-fused. From this spectroscopic evidence, the structure of **1** was concluded as 3β-nonadecanoylsitosterol (mekongsterol A).

**Table 1:** ^1^H and ^13^C NMR (500 and 125 MHz) of **1** and **2** in CDCl_3_ (*δ* in ppm, multiplicities, *J* in Hz)

Position	1		Position	2	
	*δ*_H_	*δ*_C_		*δ*_H_	*δ*_C_
1	1.15 m 1.86 m	37.2	1	1.14 m 1.86 m	37.2
2	1.84 m 1.57 m	27.9	2	1.84 m 1.57 m	27.9
3	4.62 m	73.8	3	4.62 m	73.8
4	2.30 d (7.7)	38.3	4	2.30 d (7.6)	38.3
5		139.9	5		139.9
6	5.38 d (4.4)	122.7	6	5.38 d (4.5)	122.7
7	1.98 m 1.48 m	32.0	7	1.98 m 1.48 m	32.0
8	1.44 m	32.0	8	1.43 m	32.0
9	0.95 m	50.2	9	0.95 m	50.2
10		36.7	10		36.7
11	1.00 m 1.47 m	21.1	11	1.47 m 1.00 m	21.1
12	1.20 m 2.02 m	39.9	12	1.20 m 2.02 m	39.9
13		42.5	13		42.5
14	1.07 ddd (11.9, 6.0, 5.8)	56.9	14	1.07 m	56.9
15	1.61 m 1.08 m	24.4	15	1.61 m 1.08 m	24.4
16	1.85 m 1.28 m	28.4	16	1.85 m 1.28 m	28.4
17	1.11 m	56.2	17	1.11 m	56.2
18	0.68 s	12.0	18	0.68 s	12.0
19	1.02 s	19.5	19	1.02 s	19.5
20	1.35 m	36.3	20	1.35 m	36.3
21	0.92 d (6.5)	18.9	21	0.92 d (6.5)	18.9
22	0.98 m	34.1	22	0.98 m	34.1
23	1.15 m	26.2	23	1.15 m	26.2
24	0.95 m	46.0	24	0.95 m	46.0
25	1.33 m	29.2	25	1.33 m	29.2
26	0.84 d (6.8)	19.9	26	0.84 d (6.8)	19.9
27	0.81 d (6.8)	19.2	27	0.81 d (6.8)	19.2
28	1.25 m	23.2	28	1.25 m	23.2
29	0.82 t (7.5)	12.1	29	0.84 t (7.5)	12.1
1′		173.5	1′		173.4
2′	2.27 t (7.6)	34.9	2′	2.27 t (7.6)	34.7
3′	1.62 m	25.2	3′	1.61 m	25.1
4′-17′	1.20–1.40 m	29.3–30.0	4′-6′	1.20–1.40 m	29.0
18′		22.8	7′	1.62 m	24.9
19′	0.88 t (6.9)	14.3	8′	2.34 t (7.7)	33.8
			9′		178.5

Mekongsterol B (**2**) was obtained as a white amorphous solid and showed the quasimolecular ion at *m*/*z* 607.4719 [M + Na]^+^, corresponding to the molecular formula C_38_H_64_O_4_Na in HR-ESI–MS. Absorption bands at 3500, 1710, 1730, 1600, 2960 and 2860 cm^−1^ in the IR spectrum of **2** indicated the presence of hydroxyl, acid carbonyl, ester carbonyl, double bond, methyl, methylene, and methine groups. The ^1^H NMR spectrum of **2** (Table 1) displayed signals due to two methyl singlets (*δ*_H_ 0.68, 1.02, each s), three methyl doublets (*δ*_H_ 0.81, d, *J* = 6.8 Hz; *δ*_H_ 0.84, d, *J* = 6.8 Hz; *δ*_H_ 0.92, d, *J* = 6.5 Hz), a methyl triplet (*δ*_H_ 0.84, t, *J* = 7.5 Hz), an oxymethine (*δ*_H_ 4.62, m), and trisubstituted olefinic bond (*δ*_H_ 5.38, d, *J* = 4.5 Hz), together with many aliphatic methylene and aliphatic methine groups (*δ*_H_ 0.95–2.30). The ^13^C NMR (Table 1) and DEPT spectra of **2** exhibited 38 carbons including six methyls (*δ*_C_ 12.0, 12.1, 18.9, 19.2, 19.5, 19.9), an oxymethine (*δ*_C_ 73.8), two olefinic carbons (*δ*_C_ 122.7 and 139.9), an ester carbonyl carbon (*δ*_C_ 173.4), and an acid carbonyl carbon (*δ*_C_ 178.5). These ^1^H and ^13^C data were similar to those of β-sitosterol (**3**) [9], the steroid isolated from the same extract, except for the presence of additional signals due to monoester derivative of nonadioic acid. This was confirmed by the COSY and HSQC spectra, and from them, the partial structure C(2′)H_2_–C(3′)H_2_–C(4′)H_2_–C(5′)H_2_–C(6′)H_2_–C(7′)H_2_–C(8′)H_2_ were deduced. Furthermore, the HMBC correlations from two methylene groups H_2_-2′ and H_2_-3′ to the ester carbonyl carbon C-1′, while two methylene groups H_2_-7′ and H_2_-8′ gave significant correlations to the acid carbonyl carbon C-9′ suggesting the monoester azelaic acid. The location of this moiety was determined to be at C-3 based on HMBC correlations from H-3 to C-1′ (Fig. 2). The configuration of monoester nonadioic acid moiety at C-3 to be β-equatorial orientation from the NOESY correlations H-3/H-2α and H-3/H-4α, and large *J* value (7.6 Hz) between H-3 and H-4β (Fig. 3). The relative stereochemistry of **2** was confirmed to be the same as **1** based on the results of difference NOE experiments. Thus, the structure of **2** was concluded as 3β-(8-carboxyoctanoyl)sitosterol (mekongsterol B).

### Biological assay

Among three fractions extracted from the bark of *M. Mekongensis*, *n*-hexane fraction showed α-glucosidase inhibitory activity with IC_50_ value of 17.1 µg/mL. This fraction was subjected to silica gel column chromatography to yield twelve fractions. All these fractions possessed inhibitory activity, with IC_50_ values ranging from 1.9 to 69.3 μg/mL (Table 2).

**Table 2:** α-Glucosidase inhibitory activity of fractions

Fractions	IC_50_ (µg/mL)	Fractions	IC_50_ (µg/mL)
*n*-Hexane	17.1	Fr. 6	69.3
EtOAc	>100	Fr. 7	5.1
MeOH	>100	Fr. 8	2.8
Fr. 1	21.1	Fr. 9	4.7
Fr. 2	46.8	Fr. 10	1.9
Fr. 3	39.2	Fr. 11	11.6
Fr. 4	3.9	Fr. 12	28.9
Fr. 5	4.2	Acarbose^a^	138.4

^a^Positive control

The isolated compounds were tested for their α-glucosidase inhibitory activity (Table 3). The assay was carried out at various concentrations ranging from 1 to 250 µM. Compounds **1**, **2**, **5**–**9** possessed significant α-glucosidase inhibitory activity in a concentration-dependent manner, and showed more potent inhibitory activity, with IC_50_ values ranging from 1.2 to 112.0 μM, than that of a positive control acarbose (IC_50_, 214.5 μM), which is currently used clinically in combination with either diet or anti-diabetic agents to control blood glucose level of patients [14]. Among isolated compounds, the sterol compounds (**1**–**5**) with saturated fatty ester chain or sugar group at C-3 (**1**, **2**, and **5**) showed potent α-glucosidase inhibitory activity, while the compounds with hydroxyl or ketone grop at C-3 (**3** and **4**) were inactive. On the other hand, all isolated cycloartane triterpenes (**6**–**9**) showed strong α-glucosidase inhibitory activity, however, their structure–activity relationships have not been discussed yet due to the limited number of compounds. These results indicated that the strong active compounds such as mekongsterol B (**2**; IC_50_, 2.5 μM) and magiferonic acid (**8**; IC_50_, 1.2 μM) can potentially be developed as a novel natural nutraceutical to decrease the blood glucose level because of their strong α-glucosidase inhibitory activity.

**Table 3:** α-Glucosidase inhibitory activity of the isolated compounds

Compounds	Inhibition (I %)					IC_50_ (µM)
	250 (µM)	100 (µM)	50 (µM)	25 (µM)	10 (µM)	
1	*	91.8 ± 1.1	67.7 ± 1.4	38.6 ± 1.2	24.4 ± 1.8	27.7
3	–	–	–	–	–	>250
4	–	–	–	–	–	>250
5	*	90.9 ± 1.4	75.9 ± 2.6	49.7 ± 3.1	32.1 ± 2.3	21.1
6	*	95.2 ± 2.3	85.6 ± 1.0	70.8 ± 1.2	39.0 ± 1.8	13.2
7	*	88.5 ± 1.0	75.7 ± 1.2	68.0 ± 1.1	32.9 ± 1.6	16.7
9	95.9 ± 1.0	32.2 ± 1.0	15.9 ± 1.1	–	–	112.0
Acarbose^a^	59.8 ± 1.2	21.2 ± 2.2	9.8 ± 1.1	3.2 ± 1.7	–	214.5

Compounds	Inhibition (I %)					IC_50_ (µM)
	25 (µM)	10 (µM)	5.0 (µM)	2.5 (µM)	1.0 (µM)	
2	*	93.1 ± 1.2	82.6 ± 1.4	50.8 ± 1.1	15.2 ± 1.2	2.5
8	*	87.5 ± 1.0	78.4 ± 1.6	62.5 ± 1.1	46.5 ± 1.1	1.2

* Not tested due to inessential result (IC_50_ values can be identified without these results)

– Not identified

^a^ Positive control

## Methods

### General experimental procedures

The IR spectra were measured with a Shimadzu IR-408 spectrophotometer in CHCl_3_ solution. The NMR spectra were taken on a Bruker Advance III 500 spectrometer with tetramethylsilane (TMS) as an internal standard, and chemical shifts are expressed in *δ* values. The HR-ESI–MS was performed on a Bruker MicroTOF-QII spectrometer. The absorbance (OD) was measured with a Shimadzu UV-1800 UV–Vis spectrophotometer.

### Chemicals

α-Glucosidase (EC 3.2.1.20) from *Saccharomyces cerevisiae* (750 UN) and *p*-nitrophenyl-α-d-glucopyranoside were obtained from Sigma Chemical Co. (St. Louis, MO, USA). Acarbose and dimethylsulfoxide were purchased from Merck (Darmstadt, Germany). Silica gel 60, 40–63 µm (230–400 mesh ASTM), for column chromatography was purchased from Scharlau (Barcelona, Spain). Analytical and preparative TLC were carried out on precoated Kiesegel 60F_254_ or RP-18F_254_ plates (0.25 or 0.5 mm thickness) (Merck, Germany). Other chemicals were of the highest grade available.

#### Plant material

The bark of *M. mekongensis* was collected at Ben Tre province, Vietnam, in March 2013, and was identified by Ms. Hoang Viet, Faculty of Biology, University of Science, Vietnam National University-Hochiminh City (VNU-HCMC). A voucher specimen (MDE0047) was deposited at the Division of Medicinal Chemistry, Faculty of Chemistry, University of Science, VNU-HCMC.

#### Extraction and isolation

The dried powdered bark of *M. mekongensis* (6.0 kg) was refluxed with *n*-hexane (5.0 L) in Sohxlet extractor to yield a *n*-hexane fraction (14.7 g), continuously extracted with EtOAc (5.0 L) to obtain EtOAc fraction (65.0 g), and then extracted with MeOH (5.0 L) to give MeOH fraction (108.0 g). The *n*-hexane fraction (12.5 g) was subjected to silica gel column (6.5 × 120 cm) chromatography, eluted with acetone–*n*-hexane (0–80 %) to yield 12 fractions: fr. 1 (0.1 g), fr. 2 (1.8 g), fr. 3 (1.1 g), fr. 4 (2.6 g), fr. 5 (1.4 g), fr. 6 (0.8 g), fr. 7 (0.3 g), fr. 8 (0.8 g), fr. 9 (0.7 g), fr. 10 (0.6 g), fr. 11 (0.9 g), fr. 12 (1.4 g). All extractions and fractions were tested for their α-glucosidase inhibitory activity (Table 2).

Fraction 2 (1.8 g) was applied to silica gel column chromatography with acetone-*n*-hexane gradient system to give six subfractions (fr. 2.1, 1.2 g; fr. 2.2, 134 mg; fr. 2.3, 75 mg; fr. 2.4, 47 mg; fr. 2.5, 89 mg; fr. 2.6, 270 mg). Subfraction 2.1 was chromatographed further using an CHCl_3_-*n*-hexane (0–80 %) to yield six subfractions fr. 2.1.1–6; fr. 2.1.1 (451 mg) was separated further using an EtOAc-*n*-hexane (0–30 %) to afford **1** (25.0 mg).

Fraction 4 (2.6 g) was chromatographed on silica gel column chromatography, eluted with EtOAc-*n*-hexane gradient system to give six subfractions (fr. 4.1, 717 mg; fr. 4.2, 202 mg; fr. 4.3, 993 mg; fr. 4.4, 150 mg; fr. 4.5, 78 mg; fr. 4.6, 460 mg). Subfraction 4.4 was recrystallized with MeOH-CHCl_3_ to give **4** (12.0 mg).

Fraction 5 (1.4 g) was rechromatographed to silica gel column chromatography with CHCl_3_-*n*-hexane gradient system to yield seven subfractions (fr. 5.1, 81 mg; fr. 5.2, 94 mg; fr. 5.3, 57 mg; fr. 5.4, 260 mg; fr. 5.5, 190 mg; fr. 5.6, 88 mg; fr. 5.7, 630 mg). Subfraction 5.3 was chromatographed with EtOAc-*n*-hexane (0–50 %), and then purified by normal-phase preparative TLC with CHCl_3_ (100 %) to give **3** (2.5 mg).

Fraction 6 (0.8 g) was applied to silica gel column chromatography, eluted with CHCl_3_-*n*-hexane gradient system to yield five subfractions (fr. 6.1, 124 mg; fr. 6.2, 192 mg; fr. 6.3, 272 mg; fr. 6.4, 42 mg g; fr. 6.5, 130 mg). Subfraction 6.1 was also chromatographed on silica gel with EtOAc-*n*-hexane (0–80 %), and then followed by normal-phase preparative TLC with ethyl acetate-*n*-hexane (25:75) to give **2** (8.0 mg). Subfraction 6.2 was rechromatographed further using EtOAc-*n*-hexane (0–80 %) and then purified by normal-phase preparative TLC with CHCl_3_-*n*-hexane (10:90) to give **6** (6.0 mg) and **8** (10.0 mg).

Fraction 9 (0.7 g) was chromatographed on silica gel column chromatography, eluted with CHCl_3_-*n*-hexane gradient system to give four subfractions (fr. 9.1, 150 mg; fr. 9.2, 125 mg; fr. 9.3, 360 mg; fr. 9.4, 47 mg). Subfraction 9.3 was subjected to silica gel with EtOAc-*n*-hexane (0–80 %) to yield two subfractions fr. 9.3.1–2; fr. 9.3.1 (190 mg) was separated further using a CHCl_3_-*n*-hexane (0–80 %), and then purified by normal-phase preparative TLC with EtOAc-*n*-hexane (10:90) to give **7** (6.0 mg) and **9** (10.0 mg).

Fraction 11 (0.9 g) was chromatographed on silica gel column chromatography, eluted with CHCl_3_-MeOH gradient system to give five subfractions (fr. 11.1, 42 mg; fr. 11.2, 139 mg; fr. 11.3, 93 mg; fr. 11.4, 30 mg; fr. 11.5, 570 mg). Subfraction 11.2 was subjected to silica gel with EtOAc-*n*-hexane (0–50 %) to yield two subfractions fr. 11.1.1–2; fr. 11.2.1 (60 mg) was separated further using an CHCl_3_-MeOH (0–30 %), and then purified by normal-phase preparative TLC with CHCl_3_-MeOH (96:4) to afford **5** (8.0 mg).

*Mekongsterol A* (**1**): white crystal; IR *ν*_max_ (CHCl_3_) 2950, 2870, 1720, 1610 cm^−1^; HR-ESI–MS positive *m/z* 733.6223 [M + K]^+^ (calcd for C_48_H_86_O_2_K^+^, 733.6259, error of – 3.6 mmu); ^1^H NMR (CDCl_3_, 500 MHz) and ^13^C NMR (CDCl_3_, 125 MHz), see Table 1 (For further information, see Additional file 1).

*Mekongsterol B* (**2**): white crystal; IR *ν*_max_ (CHCl_3_) 3500, 2960, 2860, 1730, 1710, 1600 cm^−1^; HR-ESI–MS positive *m/z* 607.4719 [M + Na]^+^ (calcd for C_38_H_64_O_4_Na^+^, 607.4697, error of 2.2 mmu); ^1^H NMR (CDCl_3_, 500 MHz) and ^13^C NMR (CDCl_3_, 125 MHz), see Table 1 (For further information, see Additional file 1).

#### α-Glucosidase inhibitory assay

The inhibitory activity of α-glucosidase was determined according to the modified method of Kim et al. [15]. 3 mM *p*-nitrophenyl-α-d-glucopyranoside (25 μL) and 0.2 U/mL α-glucosidase (25 μL) in 0.01 M phosphate buffer (pH = 7.0) were added to the sample solution (625 μL) to start the reaction. Each reaction was carried out at 37 °C for 30 min and stopped by adding 0.1 M Na_2_CO_3_ (375 μL). Enzymatic activity was quantified by measuring absorbance at 401 nm. One unit of α-glucosidase activity was defined as amount of enzyme liberating *p*-nitrophenol (1.0 μM) per min. The IC_50_ value was defined as the concentration of α-glucosidase inhibitor that inhibited 50 % of α-glucosidase activity. Acarbose, a known α-glucosidase inhibitor, was used as positive control.

## Conclusions

In this paper, we have reported two new compounds, mekongsterol A (**1**) and mekongsterol B (**2**), together with seven known compounds isolated from the bark of *M. mekongensis*. Seven compounds possessed α-glucosidase inhibitory activity. This is the first report on α-glucosidase inhibitory activity of the bark of this plant. These results suggested that the traditional use of the bark of *M. mekongensis* for the treatment of diabetes diseases in Vietnam may be attributable to the α-glucosidase inhibitory activity of its steroid and cycloartane constituents.

## Additional file

10.1186/s13065-016-0193-9 ^1^H, ^13^C, DEPT, COSY, HSQC, HMBC, and NOESY NMR, and MS spectra of new compounds (**1** and **2**) have been provided as an online file
