# Supplementary material for: α-Glucosidase inhibitors from the bark of Mangifera mekongensis
Source: Chem Cent J. 2016 Jul 21;10:45. doi: 10.1186/s13065-016-0193-9 (PMC4955201; doi:10.1186/s13065-016-0193-9)
Supplement: Supplementary file 1 — 10.1186/s13065-016-0193-9 1H, 13C, DEPT, COSY, HSQC, HMBC, and NOESY NMR, and MS spectra of new compounds (1 and 2) have been provided as an online file [file 13065_2016_193_MOESM1_ESM.doc]

**Supplementary data**

*α*-Glucosidase inhibitors from the bark of *Mangifera mekongensis*

Mai Thanh Thi Nguyen ^a,b^,*, Nhan Trung Nguyen ^a,b^

Affiliation

^a^ Faculty of Chemistry, University of Science, Vietnam National University Hochiminh City, Vietnam

^b^ Cancer Research Laboratory, Vietnam National University Hochiminh City, Vietnam

**Corresponding Author**

Prof. Mai Thanh Thi Nguyen, Faculty of Chemistry, University of Science, Vietnam National University-Hochiminh City, 227 Nguyen Van Cu Str., Dist. 5, Hochiminh City, Vietnam.

E-mail: nttmai@hcmus.edu.vn. Tel: 84-907-426-331

**TABLE OF CONTENTS:**

**Figure S1.1.** ^1^H-NMR spectrum of the compound **1** (500 MHz – CDCl_3_)

**Figure S1.2.** ^13^C-NMR spectrum of the compound **1** (125 MHz – CDCl_3_)

**Figure S1.3.** DEPT-NMR spectrum of the compound **1**

**Figure S1.4.** COSY-NMR spectrum of the compound **1**

**Figure S1.5.** HSQC-NMR spectrum of the compound **1**

**Figure S1.6.** HMBC-NMR spectrum of the compound **1**

**Figure S1.7.** ROESY-NMR spectrum of the compound **1**

**Figure S1.8.** HR-ESI-MS of the compound **1**

**Figure S2.1.** ^1^H-NMR spectrum of the compound **2** (500 MHz – CDCl_3_)

**Figure S2.2.** ^13^C-NMR spectrum of the compound **2** (125 MHz – CDCl_3_)

**Figure S2.3.** DEPT-NMR spectrum of the compound **2**

**Figure S2.4.** COSY-NMR spectrum of the compound **2**

**Figure S2.5.** HSQC-NMR spectrum of the compound **2**

**Figure S2.6.** HMBC-NMR spectrum of the compound **2**

**Figure S2.7.** ROESY-NMR spectrum of the compound **2**

**Figure S2.8.** HR-ESI-MS of the compound **2**

**Table S1.** ^1^H and ^13^C NMR (500 and 125 MHz) of **3**–**5** in CDCl_3_

**Table S2.** ^1^H and ^13^C NMR (500 and 125 MHz) of **6** and **8** in CDCl_3_

**Table S3.** ^1^H and ^13^C NMR (500 and 125 MHz) of **8** and **9** in CDCl_3_

**Figure S1.1.** ^1^H-NMR spectrum of the compound **1** (500 MHz – CDCl_3_)

**Figure S1.2.** ^13^C-NMR spectrum of the compound **1** (125 MHz – CDCl_3_)

**Figure S1.3.** DEPT-NMR spectrum of the compound **1**

**Figure S1.4.** COSY-NMR spectrum of the compound **1**

**Figure S1.5.** HSQC-NMR spectrum of the compound **1**

**Figure S1.6.** HMBC-NMR spectrum of the compound **1**

**Figure S1.7.** ROESY-NMR spectrum of the compound **1**


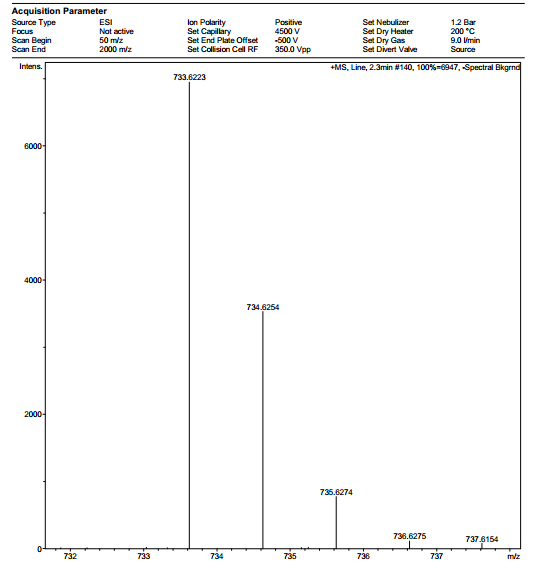


**Figure S1.8.** HR-ESI-MS of the compound **1**

**Figure S2.1.** ^1^H-NMR spectrum of the compound **2** (500 MHz – CDCl_3_)

**Figure S2.2.** ^13^C-NMR spectrum of the compound **2** (125 MHz – CDCl_3_)

**Figure S2.3.** DEPT-NMR spectrum of the compound **2**

**Figure S2.4.** COSY-NMR spectrum of the compound **2**

**Figure S2.5.** HSQC-NMR spectrum of the compound **2**

**Figure S2.6.** HMBC-NMR spectrum of the compound **2**

**Figure S2.7.** ROESY-NMR spectrum of the compound **2**


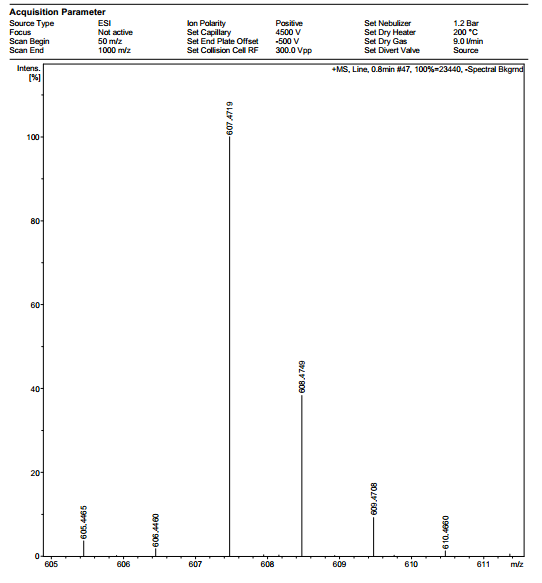


**Figure S2.8.** HR-ESI-MS of the compound **2**

Table S1. ^1^H and ^13^C NMR (500 and 125 MHz) of **3**–**5** in CDCl_3_ (*δ* in ppm, multiplicities, *J* in Hz)

| Position |  | **3** | |  | **4** | |  | **5** | |
| --- | --- | --- | --- | --- | --- | --- | --- | --- | --- |
|  |  | ***δ*_H_** | ***δ*_C_** |  | ***δ*_H_** | ***δ*_C_** |  | ***δ*_H_** | ***δ*_C_** |
| 1 |  | 1.38 (m)  1.13 (m) | 37.4 |  | 1.63 (m)  2.11 (m) | 38.1 |  | 1.85 (m)  1.14 (m) | 37.3 |
| 2 |  | 1.57 (m)  1.32 (m) | 31.8 |  | 2.57 (m)  2.33 (m) | 37.4 |  | 19.5 (m) | 29.3 |
| 3 |  | 3.53 (m) | 72.0 |  |  | 211.3 |  | 3.55 (m) | 79.6 |
| 4 |  | 2.28 (m) | 42.5 |  | 2.59 (m)  2.06 (m) | 37.0 |  | 2.27 (m) | 38.9 |
| 5 |  |  | 140.9 |  | 2.59 (m) | 57.5 |  |  | 140.3 |
| 6 |  | 5.36 (d, 4.5) | 121.9 |  |  | 209.2 |  | 5.36 (d, 4.5) | 122.2 |
| 7 |  | 1.98 (m)  1.48 (m) | 32.1 |  | 2.38 (m)  2.00 (m) | 46.6 |  | 1.98 (m)  1.47 (m) | 31.9 |
| 8 |  | 1.45 (m) | 32.1 |  | 1.85 (m) | 38.1 |  | 1.43 (m) | 31.9 |
| 9 |  | 0.95 (m) | 50.3 |  | 1.33 (m) | 53.5 |  | 0.95 (m) | 50.2 |
| 10 |  |  | 36.8 |  |  | 41.3 |  |  | 36.7 |
| 11 |  | 1.00 (m)  1.47 (m) | 21.2 |  | 1.42 (m)  1.65 (m) | 21.7 |  | 1.00 (m)  1.47 (m) | 21.1 |
| 12 |  | 1.20 (m)  2.02 (m) | 39.9 |  | 1.25 (m)  2.06 (m) | 39.4 |  | 1.20 (m)  2.02 (m) | 39.8 |
| 13 |  |  | 42.5 |  |  | 43.0 |  |  | 42.3 |
| 14 |  | 1.07 (m) | 56.9 |  | 1.07 (m) | 56.6 |  | 1.07 (m) | 56.8 |
| 15 |  | 1.61 (m)  1.08 (m) | 24.5 |  | 1.61 (m) | 24.0 |  | 1.61 (m)  1.08 (m) | 24.3 |
| 16 |  | 1.85 (m)  1.28 (m) | 28.4 |  | 1.85 (m) | 28.1 |  | 1.85 (m)  1.28 (m) | 28.2 |
| 17 |  | 1.11 (m) | 56.2 |  | 1.16 (m) | 56.0 |  | 1.11 (m) | 56.1 |
| 18 |  | 0.68 (s) | 12.0 |  | 0.69 (s) | 12.0 |  | 0.68 (s) | 11.9 |
| 19 |  | 1.01 (s) | 19.4 |  | 0.96 (s) | 12.6 |  | 1.00 (s) | 19.4 |
| 20 |  | 1.35 (m) | 36.3 |  | 1.37 (m) | 36.1 |  | 1.35 (m) | 36.2 |
| 21 |  | 0.93 (d, 6.6) | 19.2 |  | 0.93 (d, 6.5) | 18.7 |  | 0.92 (d, 6.5) | 18.8 |
| 22 |  | 0.98 (m) | 34.1 |  | 0.98 (m) | 33.8 |  | 0.98 (m) | 33.9 |
| 23 |  | 1.15 (m) | 26.2 |  | 1.16 (m) | 26.1 |  | 1.15 (m) | 26.1 |
| 24 |  | 0.92 (m) | 46.0 |  | 0.92 (m) | 45.8 |  | 0.92 (s) | 45.8 |
| 25 |  | 1.33 (m) | 29.3 |  | 1.33 (m) | 29.1 |  | 1.33 (m) | 29.1 |
| 26 |  | 0.84 (d, 6.9) | 20.0 |  | 0.84 (d, 7.2) | 19.0 |  | 0.84 (d, 6.9) | 19.8 |
| 27 |  | 0.81 (d, 6.9) | 19.6 |  | 0.81 (d, 6.8) | 19.8 |  | 0.81 (d, 6.9) | 19.0 |
| 28 |  | 1.26 (brs) | 23.2 |  | 1.26 (brs) | 23.1 |  | 1.26 (brs) | 23.1 |
| 29 |  | 0.84 (m) | 12.1 |  | 0.86 (m) | 12.0 |  | 0.84 (m) | 12.0 |
| 1' |  |  |  |  |  |  |  | 4.38 (d, 7.7) | 101.2 |
| 2' |  |  |  |  |  |  |  | 3.46 (m) | 73.6 |
| 3' |  |  |  |  |  |  |  | 3.58 (m) | 75.9 |
| 4' |  |  |  |  |  |  |  | 3.40 (d, 10.2) | 70.0 |
| 5' |  |  |  |  |  |  |  | 3.36 (m) | 74.0 |
| 6' |  |  |  |  |  |  |  | 4.42 (dd, 12.2,4.7)  4.27 (dd, 12.2,2.3) | 63.1 |
| 1'' |  |  |  |  |  |  |  |  | 174.8 |
| 2'' |  |  |  |  |  |  |  | 2.35 (t, 7.7) | 34.2 |
| 3'' |  |  |  |  |  |  |  | 1.61 (m) | 25.0 |
| 4''-13'' |  |  |  |  |  |  |  | 1.26 (brs) | 29.4 |
| 14'' |  |  |  |  |  |  |  | 1.26 (brs) | 31.9 |
| 15'' |  |  |  |  |  |  |  | 1.26 (brs) | 22.7 |
| 16'' |  |  |  |  |  |  |  | 0.88 (t, 6.9) | 14.1 |

Table S2. ^1^H and ^13^C NMR (500 and 125 MHz) of **6** and **7** in CDCl_3_ (*δ* in ppm, multiplicities, *J* in Hz)

| Position |  | **6** | |  | **7** | |
| --- | --- | --- | --- | --- | --- | --- |
|  |  | ***δ*_H_** | ***δ*_C_** |  | ***δ*_H_** | ***δ*_C_** |
| 1 |  | 1.86 (m)  1.54 (m) | 33.6 |  | 1.56 (m)  1.24 (m) | 32.1 |
| 2 |  | 2.72 (dt, 13.9,6.5)  2.32 (ddd, 14.1,4.4,2.7) | 37.6 |  | 1.76 (m)  1.56 (m) | 30.5 |
| 3 |  |  | 216.8 |  | 3.29 (m) | 79.0 |
| 4 |  |  | 50.4 |  |  | 40.6 |
| 5 |  | 1.72 (dd, 12.2,4.5) | 48.6 |  | 1.30 (m) | 47.2 |
| 6 |  | 1.58 (m)  0.80 (m) | 21.7 |  | 1.60 (m)  0.80 (m) | 21.3 |
| 7 |  | 1.33 (m)  1.08 (dd, 13.0,3.3) | 28.2 |  | 1.33 (m)  1.08 (m) | 28.2 |
| 8 |  | 1.51 (m) | 48.0 |  | 1.51 (m) | 48.1 |
| 9 |  |  | 21.2 |  |  | 20.1 |
| 10 |  |  | 26.0 |  |  | 26.2 |
| 11 |  | 1.99 (m)  1.13 (m) | 26.1 |  | 1.99 (m)  1.13 (m) | 26.1 |
| 12 |  | 1.62 (m) | 33.0 |  | 1.62 (m) | 33.0 |
| 13 |  |  | 45.5 |  |  | 45.5 |
| 14 |  |  | 48.9 |  |  | 49.0 |
| 15 |  | 1.32 (m) | 35.7 |  | 1.30 (m) | 35.7 |
| 16 |  | 1.88 (m)  1.28 (m) | 26.9 |  | 1.88 (m)  1.28 (m) | 26.6 |
| 17 |  | 1.60 (m) | 52.4 |  | 1.61 (m) | 52.4 |
| 18 |  | 0.96 (s) | 19.5 |  | 0.96 (s) | 19.5 |
| 19 |  | 0.57 (d, 4.3)  0.78 (d, 4.3) | 29.7 |  | 0.55 (d, 4.1)  0.33 (d, 4.2) | 30.1 |
| 20 |  | 1.44 (m) | 36.2 |  | 1.42 (m) | 36.2 |
| 21 |  | 0.89 (d, 6.3) | 18.2 |  | 0.89 (d, 6.3) | 18.2 |
| 22 |  | 1.64 (m)  1.19 (m) | 34.7 |  | 1.62 (m)  1.18 (m) | 34.6 |
| 23 |  | 2.17 (m)  1.95 (m) | 31.8 |  | 2.17 (m)  1.95 (m) | 31.7 |
| 24 |  |  | 148.7 |  |  | 148.7 |
| 25 |  | 3.17 (q, 7.1) | 45.6 |  | 3.18 (q, 7.0) | 45.6 |
| 26 |  |  | 179.1 |  |  | 179.0 |
| 27 |  | 1.30 (d, 7.3) | 16.5 |  | 1.31 (d, 7.1) | 16.5 |
| 28 |  | 0.91 (s) | 18.4 |  | 0.96 (s) | 18.4 |
| 29 |  | 1.05 (s) | 20.4 |  | 0.81 (s) | 25.6 |
| 30 |  | 1.10 (s) | 22.3 |  | 0.89 (s) | 14.2 |
| 31 |  | 4.97 (brs)  4.93 (brs) | 111.3 |  | 4.96 (brs)  4.92 (brs) | 111.3 |

Table S3. ^1^H and ^13^C NMR (500 and 125 MHz) of **8** and **9** in CDCl_3_ (*δ* in ppm, multiplicities, *J* in Hz)

| Position |  | **8** | |  | **9** | |
| --- | --- | --- | --- | --- | --- | --- |
|  |  | ***δ*_H_** | ***δ*_C_** |  | ***δ*_H_** | ***δ*_C_** |
| 1 |  | 1.86 m  1.54 m | 33.6 |  | 1.56 m  1.25 m | 32.0 |
| 2 |  | 2.70 ddd (13.9, 13.9, 6.4)  2.30 ddd (13.9, 4.1, 2.6) | 37.6 |  | 1.75 m  1.56 m | 30.4 |
| 3 |  |  | 216.7 |  | 3.30 dd (11.2, 4.4) | 78.9 |
| 4 |  |  | 50.4 |  |  | 40.5 |
| 5 |  | 1.72 dd (12.2, 4.4) | 48.6 |  | 1.30 m | 47.1 |
| 6 |  | 1.55 m  0.94 m | 21.7 |  | 1.61 m  0.78 m | 21.1 |
| 7 |  | 1.37 m  1.14 m | 26.1 |  | 1.33 m  1.08 m | 26.0 |
| 8 |  | 1.59 m | 48.0 |  | 1.52 dd (12.3, 4.6) | 47.9 |
| 9 |  |  | 21.3 |  |  | 20.0 |
| 10 |  |  | 26.2 |  |  | 26.1 |
| 11 |  | 2.05 m  1.16 m | 26.9 |  | 2.00 m  1.14 m | 26.5 |
| 12 |  | 1.66 m | 33.0 |  | 1.63 m | 32.9 |
| 13 |  |  | 45.6 |  |  | 45.4 |
| 14 |  |  | 48.9 |  |  | 48.8 |
| 15 |  | 1.32 m | 35.7 |  | 1.30 m | 35.5 |
| 16 |  | 1.91 m  1.31 m | 28.3 |  | 1.89 m  1.28 m | 28.1 |
| 17 |  | 1.61 m | 52.4 |  | 1.58 m | 52.2 |
| 18 |  | 1.00 s | 18.2 |  | 0.96 s | 18.0 |
| 19 |  | 0.79 d (4.0)  0.57 d (4.0) | 29.7 |  | 0.56 d (4.0)  0.34 d (4.0) | 29.9 |
| 20 |  | 1.43 m | 36.1 |  | 1.44 m | 36.0 |
| 21 |  | 0.92 d (6.6) | 18.3 |  | 0.91 d (6.5) | 18.1 |
| 22 |  | 1.58 m  1.17 m | 35.0 |  | 1.58 m  1.18 m | 34.8 |
| 23 |  | 2.26 m  2.12 m | 26.0 |  | 2.25 m  2.12 m | 25.9 |
| 24 |  | 6.91 t (7.6) | 145.8 |  | 6.90 t (7.3) | 145.7 |
| 25 |  |  | 126.8 |  |  | 126.7 |
| 26 |  |  | 173.0 |  |  | 172.9 |
| 27 |  | 1.84 s | 12.1 |  | 1.84 s | 11.9 |
| 28 |  | 1.05 s | 22.4 |  | 0.81 s | 25.4 |
| 29 |  | 1.10 s | 20.9 |  | 0.97 s | 14.0 |
| 30 |  | 0.91 s | 19.5 |  | 0.90 s | 19.3 |
